# Differential diagnosis of a thyroid mass, facial malar rash and ptosis on the flora in the primavera by Sandro Botticelli (1445–1510)

**DOI:** 10.1007/s40618-021-01623-3

**Published:** 2021-07-09

**Authors:** H. Ashrafian

**Affiliations:** The Department of Surgery and Cancer, St Mary’s Hospital, Institute of Global Health Innovation, Imperial College London, 10th Floor Queen Elizabeth the Queen Mother (QEQM) Building, Praed Street, London, W2 1NY UK

**Keywords:** Thyroid, Goiter, SLE, Lupus, Rash, Malar, Autoimmunity, Pregnancy

## Abstract

**Purpose:**

The Primavera is considered amongst the greatest and controversial artistic masterpieces worldwide painted by renaissance artist Sandro Botticelli. The aim was to identify any underlying medical foundations for the painting.

**Methods:**

Observational study.

**Results:**

The painting reveals, a ‘butterfly’ malar rash, bilateral ptosis and a clear neck swelling consistent with a goitre in the figure of Flora. This could be explained by concomitant Graves’ disease and systemic lupus erythematosus, or other presentations of multiple autoimmune syndrome.

**Conclusion:**

These findings highlight the likely presentation of the earliest pictorial depictions of thyroid disease with systemic lupus erythematosus and emphasize the exactitude of depiction demonstrated by Botticelli in renaissance era.

The Primavera or ‘Spring’ painted during the Early Renaissance (circa late 1470–early 1480) by Sandro Botticelli (Fig. 1a) is considered amongst the greatest and controversial masterpieces worldwide. Its notoriety stem from its allegorical nature of the season of Spring, fertility and breaking of chastity presented through classical characters including Venus, the Three Graces, Mercury, Zephyrus the West Wind and Chloris the goddess of flowers (in addition to previous identification of discernible anatomy such as the structure of lungs in the background tree line and the possibility of a goitre in the Venus character).

**Fig. 1:**
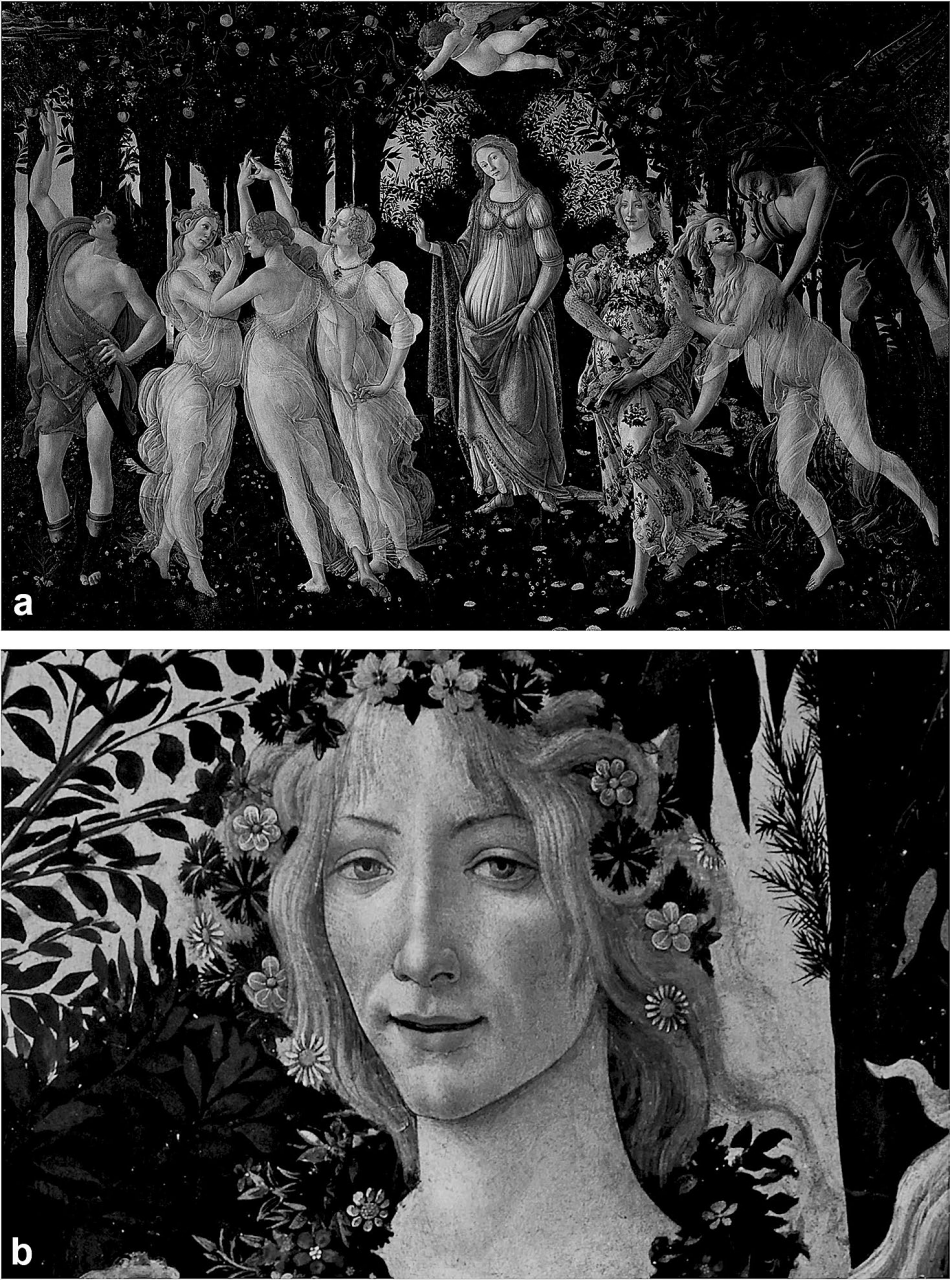
The Primavera (1477–1482) by Sandro Botticelli, **a** complete painting **b** close-up of Flora (The Goddess of Spring) © Uffizi Gallery, Florence, Italy

I note that the figure of Flora (or spring) demonstrates a clear neck swelling consistent with a goiter (Fig. 1b) with bilateral ptosis and facial flushing consistent with a ‘butterfly’ malar rash and the possibility of periorbital heliotrope rash more prominent around the right eye. This likely derives from the real-time findings in one of the models utilised by Botticelli during the painting of the piece.

Differential diagnosis of the goiter with ptosis and the malar rash could represent the individual depicted may have suffered from concomitant thyroid and malar-causing autoimmunity. This could be explained by concomitant Graves’ disease (GD) and malar-associated systemic lupus erythematosus (SLE) (where the prevalence of GD in SLE is 68 × higher than the general population) or Hashimoto’s thyroiditis (HT) and SLE (where the prevalence of HT in SLE is 90 × higher than the general population) [1]. The presence of ptosis would favour a diagnosis of Graves’ disease with associated ophthalmopathy and ocular myasthenia. These could represent multiple autoimmune syndromes as more than three distinct autoimmune conditions co-occur, and additionally a fourth could be dermatomyositis seen in the possibility of a heliotrope rash. As the subject in the painting is depicted as pregnant, which may have represented an accurate representation, then these findings could correspond to the increased prevalence of autoimmunity in pregnancy [2] where some autoimmune conditions such as rheumatoid arthritis are known to subside and others remain symptomatically unchanged or even exacerbated as in lupus; where there are also increased obstetric complication rates. A rare though notable differential would also include the facial rash being that of facial flushing secondary to thyroid carcinoma, a finding most associated medullary thyroid cancer.

Further support of Botticelli’s allegory of spring in this piece could include the finding that if the SLE was pathology in the character of Flora, there is seasonal evidence that this condition is more prevalent in Spring, and most conspicuously the malar rash is most identifiable in this season. These findings highlight the depth of genius in this great artist and strengthen the understanding of scientific principles captured in the magnificent renaissance artistic portrayals.
